# Inkjet Printing of Colloidal Nanospheres: Engineering the Evaporation-Driven Self-Assembly Process to Form Defined Layer Morphologies

**DOI:** 10.1186/s11671-015-1065-2

**Published:** 2015-09-16

**Authors:** Enrico Sowade, Thomas Blaudeck, Reinhard R. Baumann

**Affiliations:** Digital Printing and Imaging Technology, Technische Universität Chemnitz, 09126 Chemnitz, Germany; Center for Microtechnologies, Technische Universität Chemnitz, 09107 Chemnitz, Germany; Department Printed Functionalities, Fraunhofer Institute for Electronic Nanosystems, 09126 Chemnitz, Germany

**Keywords:** Inkjet printing, Self-assembly, Colloidal suspension

## Abstract

We report on inkjet printing of aqueous colloidal suspensions containing monodisperse silica and/or polystyrene nanosphere particles and a systematic study of the morphology of the deposits as a function of different parameters during inkjet printing and solvent evaporation. The colloidal suspensions act as a model ink for an understanding of layer formation processes and resulting morphologies in inkjet printing in general. We investigated the influence of the surface energy and the temperature of the substrate, the formulation of the suspensions, and the multi-pass printing aiming for layer stacks on the morphology of the deposits. We explain our findings with models of evaporation-driven self-assembly of the nanosphere particles in a liquid droplet and derive methods to direct the self-assembly processes into distinct one- and two-dimensional deposit morphologies.

**Electronic supplementary material:**

The online version of this article (doi:10.1186/s11671-015-1065-2) contains supplementary material, which is available to authorized users.

## Background

Self-assembly processes of molecules or micro- or nanoscopic particles within droplets are an interesting method for the development of ordered assemblies and have attracted considerable interest during the last decades. During the evaporation of the solvents of the droplet, different transport mechanisms force the molecules or particles to certain positions where they assemble and form in part rigid agglomerates. Such explained self-assembly processes are ubiquitous, a natural phenomenon [1–3], and considered also as a promising tool for nanofabrication [4–6]. The resulting deposits, their morphology, and functional properties are very important for many applications, e.g., printing and coating technologies such as inkjet printing, spin coating, or slot-die coating, for paintings and coatings, for bioassay manufacturing, and many others [1, 7–9].

Employing self-assembly processes for molecules or particles within an evaporating patterned or non-patterned liquid film has turned out to be a simple and elegant method to achieve a packing of the dispersed constituents, e.g., for the assembly of ordered structures from colloidal particles in droplets [10–13], one-dimensional lines [14, 15], two-dimensional patterned photonic crystals [16], or even three-dimensional spherical colloidal assemblies [17]. However, numerous publications show the complexity of the fluid dynamics which the particles and constituents undergo aside of Brownian motion [18], gravity [19], and buoyancy during the evaporation. Phenomena such as capillary flows [20], convectional flows [21], and nonetheless the attractive (e.g., van der Waals) and repulsive forces (1) amongst particles [8, 16], (2) between particles and substrate [15, 22, 23], and/or (3) at the three-phase boundary (line-tension effects) [19, 24] will contribute to the final shape of the deposit. The deposits obtained by an evaporating droplet containing nanoparticles on non-absorbent and rigid surfaces can range from uniform patterns [11] to a ring-like pattern via the so-called coffee ring effect [20], central bumps [21], and inner coffee ring deposits [22] or and a number of further patterns in between [9].

There are two transport mechanisms in literature studied most: (1) capillary flows transporting materials and particles from the center towards the edge where they accumulate due to a pinning of the contact line and (2) inward flows from the droplet edge to the center usually leading to central bumps [2, 8]. For these cases, detailed theoretical and experimental investigations have been made trying to explain the droplet impact, droplet evaporation, and the resulting shape of the deposit [8, 25]. It has been demonstrated that these two transport mechanisms are also of high interest for inkjet printing as well as other direct-writing technologies since they define the morphology of the printed layer and its properties [26–28].

In this research work, following earlier studies on inkjet-printed self-assembled molecular monolayers and self-assembled spherical colloidal assemblies [17, 29], the influence of different parameters on the inward- and outward-directed transport flows of inkjet-printed sessile colloidal droplets on non-absorbent surfaces is investigated focusing on the morphology of deposit. We combine the approach of bottom-up manufacturing based on self-assembly with inkjet printing, a flexible, scalable, and direct-writing deposition technique. Thus, we are presenting a systematic study of the resulting deposits of the inkjet-printed colloidal suspensions as a function of surface energy of the substrate, the temperature of the substrate, and the ink formulation. The monodisperse nanosphere particles of the suspensions serve as a model system for understanding the self-assembly phenomena.

## Methods

Different commercially available colloidal suspensions were applied as inks for inkjet printing. The commercial suspensions contain (1) highly monodisperse organic polystyrene (PS) nanosphere particles with an anionic, hydrophobic surface or (2) inorganic silica (Si) nanospheres with non-functionalized polar hydroxyl surface groups (Si-OH), both suspended in an aqueous solvent base. The colloidal inks were obtained from BS-Partikel GmbH, Duke Scientific (Palo Alto, CA, USA) and Bangs Laboratories (Fishers, IN, USA). Details of the used colloidal suspensions and their characteristics are provided in Table 1. The surface tension of the suspensions was determined using a DataPhysics OCA20 (DataPhysics Instruments GmbH, Filderstadt, Germany) system in pendant drop mode. The pH value was determined with universal indicator paper (Munktell, Bärenstein, Germany and Macherey Nagel, Düren, Germany). All the colloidal suspensions were treated ultrasonically for about 3–15 min before printing. No further filtering process as usual for inkjet printing was done since the particles are monodisperse, and ultrasonic treatment was applied to re-disperse and re-agglomerate the nanospheres.

**Table 1:** Characteristics of the colloidal suspensions used for the experiments

Manufacturer	BS-Partikel (BS305)	Duke Scientifics (DS300)	Bangs Laboratories (BL280)
Nanosphere material	Polystyrene	Polystyrene	Silica
Nanosphere diameter (nm)	305 ± 8	300 ± 5	280 ± 7
Solids content (wt.%)	2	0.1	2
Surface tension (mN/m)	46.8 ± 0.8	57.3 ± 0.9	70.2 ± 1.5
pH value	7.0 ± 0.2	7.0 ± 0.2	7.0 ± 0.2

Differently treated cover slip glasses (18 × 18 mm^2^ and 20 × 20 mm^2^; thickness 0.145 ± 0.015 mm, purchased from VWR Scientific, Dresden, Germany) were employed as substrates for the printing process. Following treatments were applied to vary the surface energy of the cover slip glasses and thus the contact angle of a sessile droplet of water with the glasses (see Table 2): (1) “untreated,” only cleaned with ethanol; (2) hexamethyldisilazane (HMDS) treatment; (3) octadecyltrichlorosilane (OTS) treatment; (4) surfactant treatment (based on anionic surfactants); and (5) corona treatment. The contact angle on all surfaces was determined with pure deionized water droplets (resistivity about 16 MΩ·cm) using the DataPhysics OCA20 system in sessile drop mode. The surfactant treatment and the silane treatments were done in a chemical bath according to known methods. Corona treatment was performed using an Arcotec corona generator CG061-2 (Arcotec GmbH, Mönsheim, Germany) with a high-voltage discharge of 2.3 kV.

**Table 2:** Measured contact angle of sessile droplets of deionized water on the differently treated glass substrates

	Corona treatment	Surfactant treatment	Untreated	HMDS treatment	OTS treatment
Contact angle (°)	<10	<10	67.7 ± 2.7	78.7 ± 1.5	100 ± 5

The colloidal suspensions were printed using a Dimatix DMP 2831 laboratory drop-on-demand (DoD) inkjet printer (Fujifilm Dimatix Inc., Santa Clara, USA). The inkjet printheads have a nozzle diameter of 21.5 μm and a nominal drop volume of 10 pL. The DMP was applied in both single nozzle and multi nozzle modes. The clear distance between the nozzle and the substrate was maintained at 1 mm during printing. All samples were printed at ambient conditions (laboratory conditions 22.5 ± 0.8 °C and 22 ± 3 % relative humidity).

The printed deposits were analyzed by scanning electron microscopy (SEM) using a Hitachi TM-1000 (Hitachi High-Technologies Cooperation, Tokyo, Japan). To avoid the charging effect on the insulating nanospheres, the samples were coated with an about 18-nm-thick layer of Pt by sputtering at 40 mA for 120 s using a BAL-TEC SCD 050 (formerly BAL-TEC AG, Balzers, Liechtenstein) electron microscope preparation system. Optical microscopy analysis was carried out on a Leica DM 4000 M (Leica Microsystems CMS GmbH, Wetzlar, Germany).

## Results and Discussion

### Influence of Substrate Surface Energy on Droplet Deposit Morphology

**Fig. 1:**
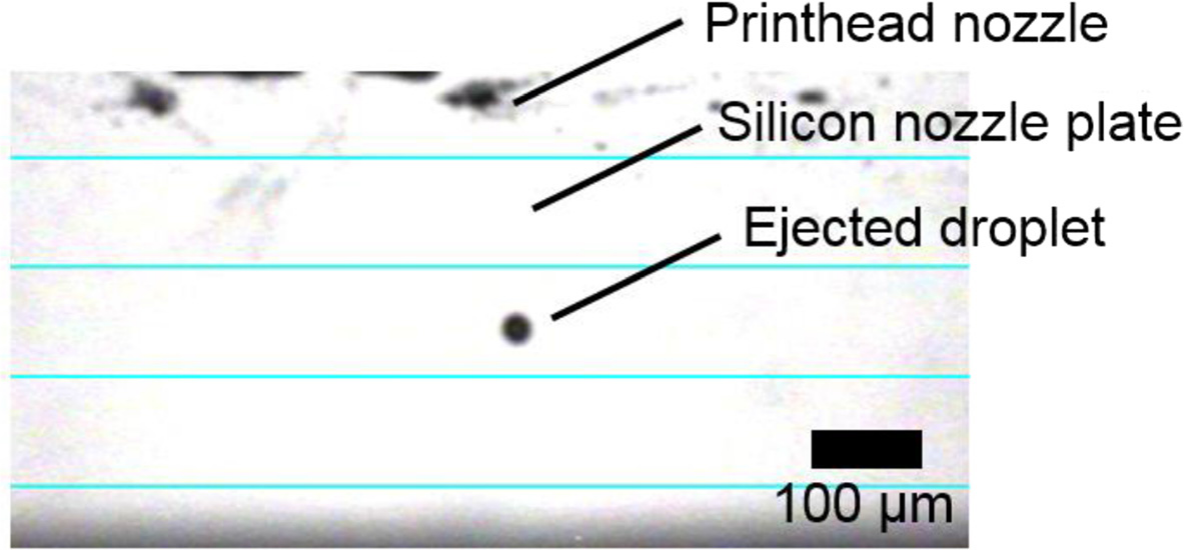
Drop-watcher camera image of an ejected droplet of BS305 with the DMP 2831 inkjet printer

Parameters of the inkjet printing system such as voltage applied to the piezoelectric transducer and the meniscus pressure were optimized towards a stable ejection of a ball-shaped droplet of the colloidal suspensions with a jetting frequency of up to 5 kHz. Figure 1 shows an image of an ejected droplet of BS305 using the drop-watcher camera of the inkjet printing system. The ejected droplet is well-defined, ball-shaped, and without tail or satellite droplets indicating a stable jetting process. Only one nozzle was used for the experiments in the following sections. It was found that the droplet jetting process became more instable with increase in weight percentage of particles in the ink formulation resulting in nozzle clogging. Cleaning cycles were introduced during the printing based on a purge process to prevent nozzle clogging or to remove the clogging.

**Fig. 2:**
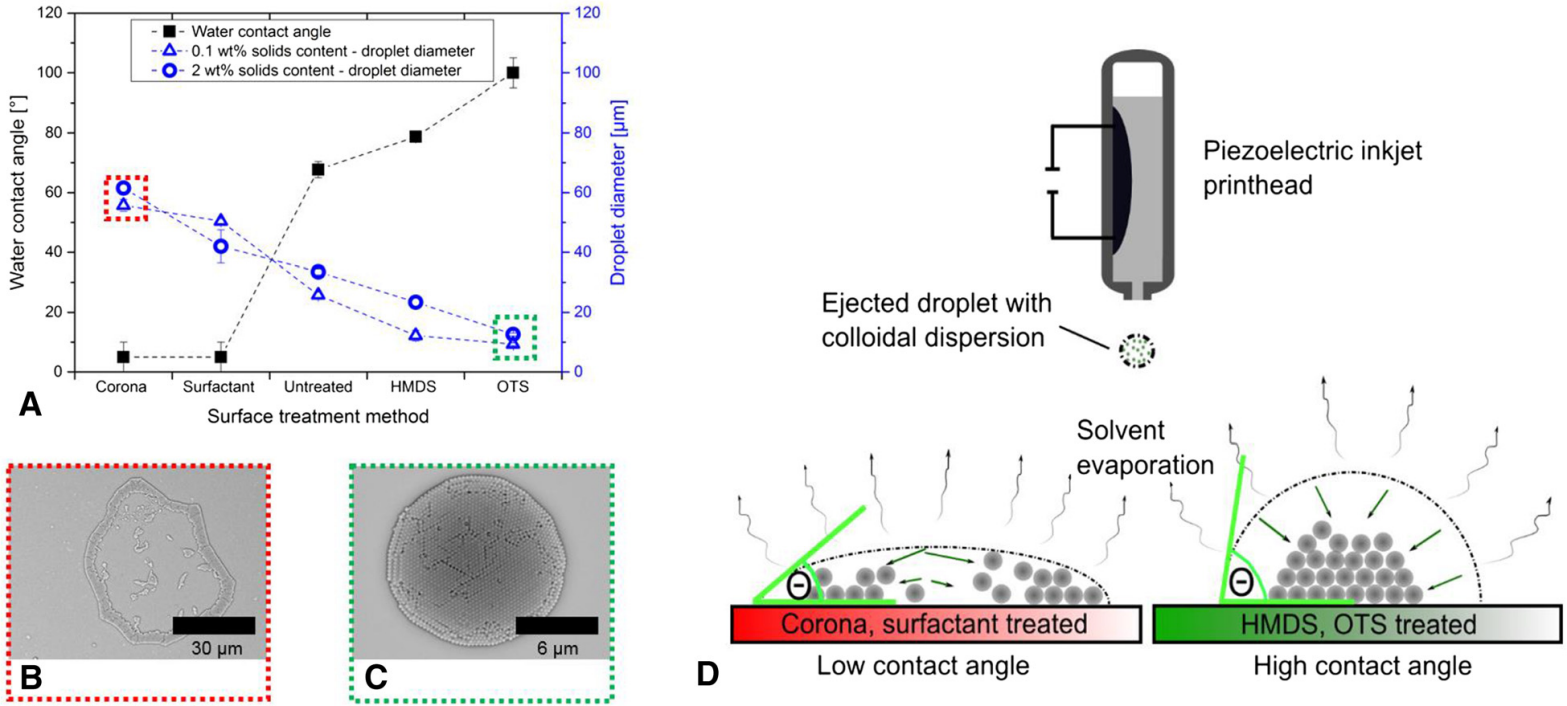
**a** Measured water contact angle and measured droplet diameter of the deposits obtained by printing of a 0.1 wt.% colloidal suspension DS300 and a 2.0 wt.% colloidal suspension BS305 as a function of surface treatment method. **b**, **c** SEM images of typical deposits of the highlighted samples of (**a**) with 2 wt.% solids content. **d** The principle morphology of the deposits as a function of contact angle of the deposited droplet with the substrate

Figure 2a shows a plot of the water contact angle and droplet deposit diameter as a function of the differently treated substrates using the 0.1 wt.% colloidal suspension DS300 and the 2.0 wt.% colloidal suspension BS305. SEM images of characteristic droplet deposits on all the differently treated substrates are available in Additional file 1: Figure S1. Exemplarily, a droplet deposit is depicted in Fig. 2b, c on corona-treated glass substrate and OTS-treated glass substrate. The principle of the formation of a droplet deposit as a function of the surface energy of the substrate characterized by the static water contact angle is shown in the scheme in Fig. 2d.

As it can be seen from Fig. 2a, it turns out that the higher the water contact angle on the substrates, the lower the diameter of the resulting droplet deposit is. The surface treatment also strongly affects the morphology of the deposit by virtue of the packing of the nanospherical constituents as indicated in Fig. 2b, c as well as in Additional file 1: Figure S1. At low water contact angles (high surface energy of the substrate), resulting capillary flows will transport the nanospheres from the center of the evaporating droplet towards the edge where they agglomerate. This phenomenon is described as coffee ring effect and well observed, investigated, and sometimes also exploited for certain applications many times in literature [5, 8, 20, 27, 28]. At high contact angles (low surface energy of the substrate), no coffee ring effect can be seen but the nanosphere particles are stacked in multilayers. Even colloidal hemispheres (printed domes) can be obtained by printing or dispensing if the substrate has a very low surface energy enabling a high receding contact angle and thus a freely sliding three-phase contact line (see Additional file 1: Figure S2) [10–12, 30]. In our experiments, the area coverage with nanosphere particles of single, isolated inkjet droplets was calculated on the basis of the SEM images by determining the ratio of the number of pixels circumscribed by the deposited particles and the number of pixels making up the total droplet footprint marking the deposit area. Alike in the previous case, it turns out here that the lower the water contact angle and the higher the spreading of the inkjet-printed droplet on the substrate, the lower the particle area coverage is (see Additional file 1: Figure S3). A coverage of 100 % was obtained for BS305 on HMDS- and OTS-treated substrates indicating a continuously covered area with at least one monolayer of nanospheres.

Aside of the morphology, the substrate properties also affect the circularity of the deposits. An almost ideal circularity with a very low eccentricity of about 0.2 μm was obtained on HMDS- and OTS-treated substrates whereas deviations from the circularity become more obvious for the deposits on corona- and surfactant-treated glasses. In our experiments, the drop-to-drop variation in diameter was determined for 20 sequentially printed droplets of BS305 on OTS-treated substrates. The drop diameter was about 12.6 μm and the drop-to-drop variation in diameter about 4 %. The influence of the substrate treatment method on the morphology of the droplet deposit is summarized qualitatively in Table 3.

**Table 3:** Qualitative summary of main trends based on correlations between printing results and varying printing parameters

Water contact angle	Diameter of the deposits	Circularity of the deposits	Number of layers of the nanospheres	Degree of manifestation of coffee ring effect	Particle area coverage of the circumscribed deposit area
Low	High	Low	Low	High	Low
High	Low	High	High	Low	High

### Counteracting the Coffee Ring Effect and Printed Layer Stacks

Aside of the usage of glass substrates with an high contact angle, one can also counteract the coffee ring effect by tuning the ink composition, e.g., by adding high boiling point solvents [11, 31, 32] to decrease the evaporation rate. However, the evaporation behavior becomes very complex for these kinds of co-solvent/surfactant/particle substrate systems since they are considered as non-equilibrium processes [9]. Surfactants are mainly part of the polymer nanosphere suspensions because they are incorporated during the emulsion polymerization process (see surface tension measurement in Table 1, high surface tension indicates low amount of surfactants and vice versa [33]). It has been reported that the dissolved surfactants may absorb on the substrate and on the nanospheres as well as on the air/solution interface resulting in alteration of the initial contact angle and contact area of the sessile droplet. Surfactants also affect the pinning conditions of the three-phase contact line and all factors finally influence the deposit morphology [9, 33]. The co-solvent which we will add strongly influences the evaporation rate of the droplet which is considered one of the main factors for the ordering of the particles [34]. It was found that electrostatic repulsion and van der Waals forces play a minor role for ordering since the attractive capillary forces upon meniscus formation between solvent and nanospheres during evaporation dominate [9, 34].

**Fig. 3:**
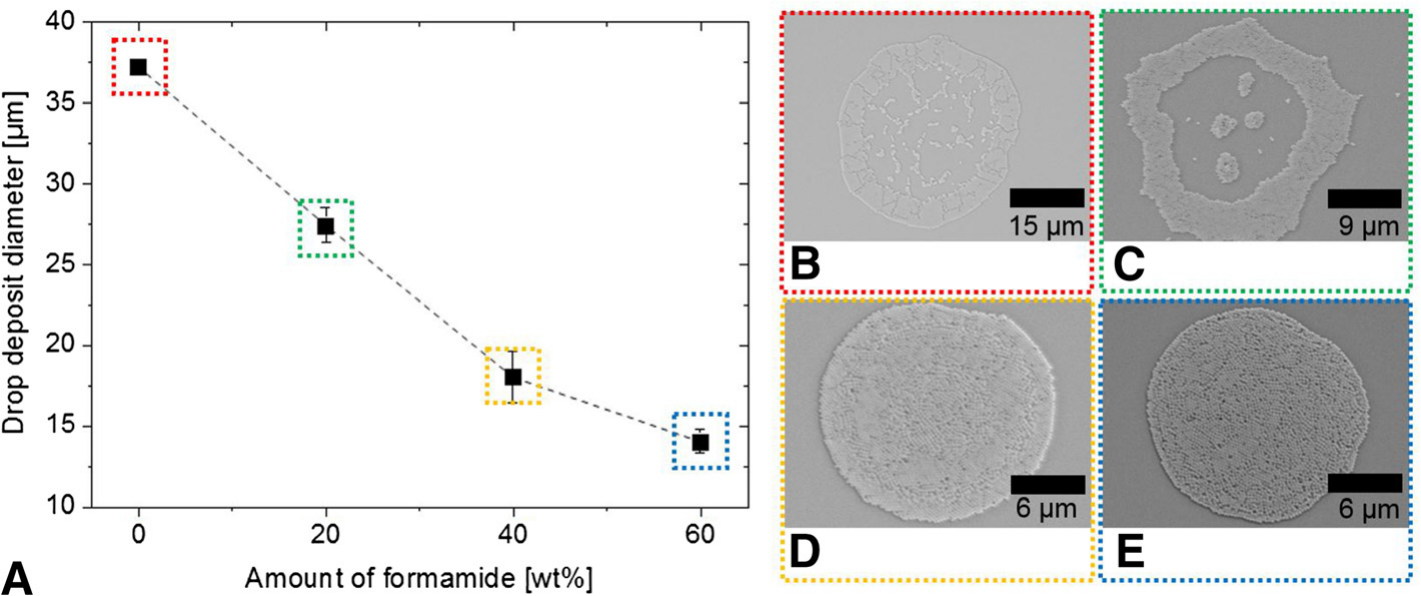
**a** Diameter of the droplet deposit as a function of the amount of formamide in the ink formulation; **b**–**e** SEM images of typical deposits of the highlighted samples of (**a**). The amount of formamide is increasing from (**a**) to (**b**) and the droplet morphology changes accordingly

Figure 3 shows the decrease of the drop diameter as a function of the amount of formamide (boiling point 210 °C, vapor pressure 0.07999 mmHg at 25 °C) added to the colloidal suspension (on untreated glass). The solids content is in all cases 2 wt.% and silica nanospheres were used. Addition of formamide increases the evaporation time of the deposited droplets and reduces gradually the coffee ring effect as depicted in the SEM images in Fig. 3b–e. Without formamide, an intense spreading, and finally, a contact line pinning of the immobilized droplets are observed. These facts result in a comparable large droplet diameter with the significant coffee ring morphology and the formation of a monolayer (Fig. 3b). The higher the amount of formamide, the lower the drop diameter. The coffee ring structure is not obtained for 40 wt.% formamide or higher in the ink formulation. This observation can be explained by induced Marangoni flows, which counteract the convective flows leading to the coffee ring effects. Details of the phenomenon are well explained and investigated by Hu et al. [21], De Gans et al. [31], Jeong et al. [32], and Park et al. [11]. However, one has to consider also a change in surface tension and viscosity of the ink formulation when adding co-solvents such as formamide resulting finally in different drop deposit diameters and morphologies. This fact has not been addressed before but it is important because a change in droplet diameter usually implies also a change in print resolution in the case of printed lines or patterns that are even more complex.

**Fig. 4:**
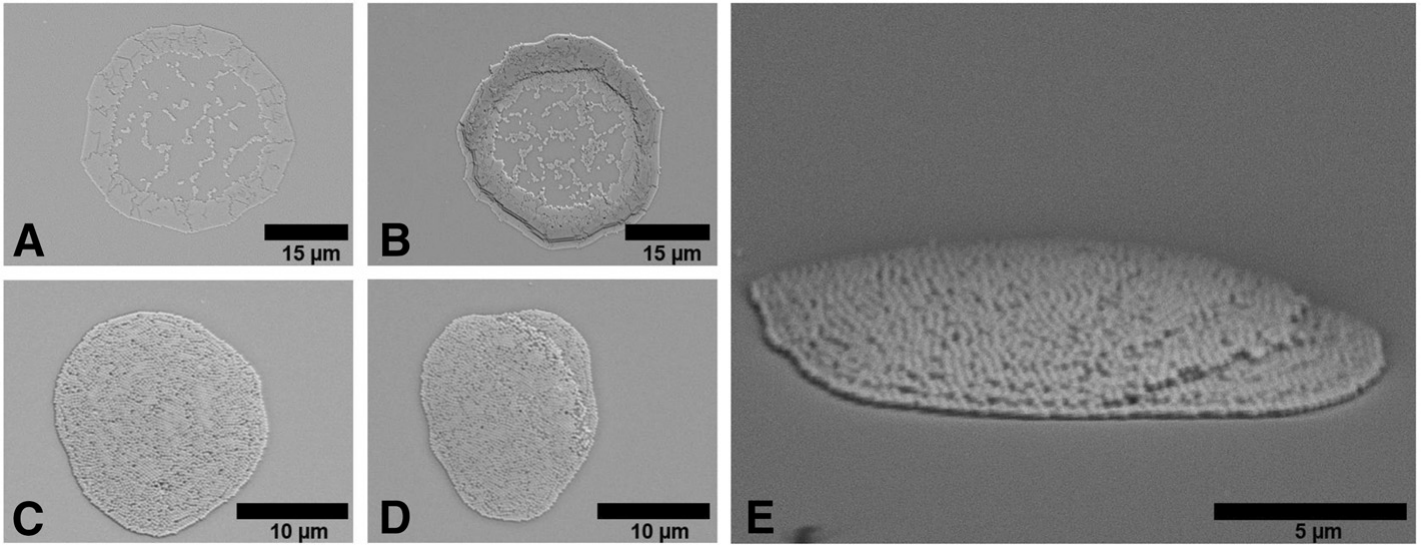
Deposits of inkjet-printed colloidal suspension on cleaned glass slides; the deposit **a** was obtained with one droplet of BS305 and **b** with printing two droplets of the ink on top of each other; **c** was obtained with one droplet of BL280 with 2 wt.% silica and 60 wt.% formamide and **d** with printing two droplets of the ink on top of each other; **e** is a side view of (**d**) at about 85° substrate inclination

Multilayer stacks can be obtained by the deposition of a second droplet on an already deposited and dried droplet. They are interesting, e.g., for the development of photonic crystals based on parallel stacks of layers of different dielectric constant forming a photonic band gap in a single direction. These one-dimensional photonic crystal structures can be applied as nanoparticle-based Bragg reflectors [35]. One further point of interest is the question of the adhesion of the nanospheres to the substrate and among each other. If the adhesion is low, the solvent of the second droplet impinging in a wet-on-dry multi-pass printing set might re-disperse the deposited particles and rearrange them, e.g., in a different morphology. Figure 4 shows deposits of the experiment of printing a second drop of an already dried drop deposit. Figure 4a (2 wt.% BS305 ink formulation) and Fig. 4c (2 wt.% BL280 ink formulation with 40 wt.% formamide) represent single droplets printed on cleaned glass with and without the coffee ring morphology. After the deposition of the first droplet, the samples were kept on the substrate holder of the printer for several minutes to allow the complete evaporation of the solvent. Then, the same ink formulations were printed as single droplets on top of the dried deposits. Figure 4b, d depicts the result. Figure 4e is a side view of Fig. 4d at about 85° substrate inclination. The printing process and thus the drop ejection have a high accuracy since the second droplet was deposited at exactly the same position of the first droplet. The second droplets do not seem to dissolve and re-disperse the underlying nanospheres but establish a clearly distinguishable second layer on the first. The morphology of the second deposit follows well the morphology of the first deposit. The coffee ring becomes more pronounced in Fig. 4b due to the assembly of most of the nanospheres of the second drop on the already existing coffee ring deposit. Although the evaporation time and thus the available time for re-dispersing nanospheres is increased for the ink formulation with formamide (higher boiling point), the second drop seems to form a separate layer without any dissolving of the first deposit as indicated by Fig. 4d, e. Thus, the adhesion between the individual nanospheres and the nanospheres and the substrate can be considered as very high.

### Influence of Substrate Temperature on Droplet Deposit

**Fig. 5:**
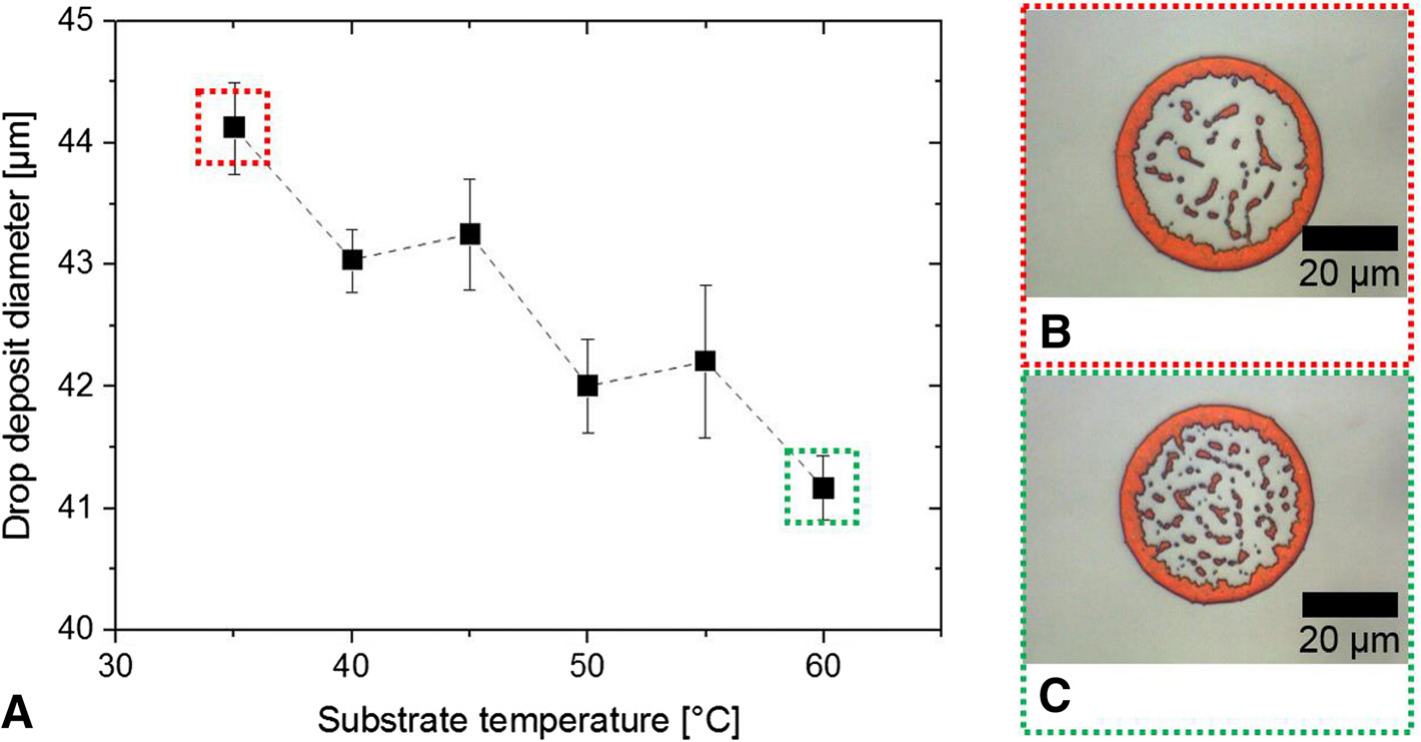
**a** Diameter of the droplet deposit as a function of temperature applied to the substrate; **b**, **c** reflected light microscopy images of typical deposits of the highlighted samples of (**a**)

Another important parameter to influence the deposit morphology of printed droplets next to the wettability of the substrate and the ink formulation is the temperature of the substrate [36, 37]. Figure 5a shows the measured diameters of the droplet deposits of BS305 diluted to 1 wt.% polystyrene particles on glass cleaned in ultrasonic bath with acetone and ethanol. The temperature of the substrates was varied starting from 35 to 60 °C. A higher substrate temperature contributes obviously to lower deposit diameters. The solvent evaporates faster preventing excessive spreading of the deposited droplet on the substrate. Figure 5b–c is images captured by reflected light microscopy. Due to the interaction of the clusters of the ordered nanospheres with the light, the deposits appear red. The nanospheres are usually packed in a face-centered cubic (fcc) lattice with the (111) plane parallel to the substrate [10]. According to Bragg’s law, the corresponding wavelength *λ* can be calculated by

1 $$ \lambda =2\times 0.816\times {n}_{\mathrm{eff}}\times d $$

where *λ* is the wavelength, *n*_eff_ is the effective refractive index of the nanospheres with the environmental air, and *d* is the diameter of nanosphere. According to this equation, the peak wavelength should be observed at about 715 nm assuming a refractive index of the particles of 1.4366. This also corresponds to the red color of the deposits in Fig. 5b–c.

There is not a very big difference comparing the deposit morphology at 35 °C substrate temperature (Fig. 5b) and 60 °C substrate temperature (Fig. 5c). The coffee ring effect takes place at both conditions indicating a pinned contact line. The extension of the contact line is limited in the case of high substrate temperature so that the deposit diameter is finally less. Compared to the deposit at 35 °C, a higher amount of particles inside the coffee ring at 60 °C is remarkable. This observation usually indicates that the coffee ring effect is less pronounced compared to droplet samples without particles in the center. However, we assume that the convective flows resulting in the coffee ring deposit were interrupted due to the high temperature of the substrates. The solvent evaporation of a sessile droplet on the substrate with a nominal volume of 10 pL takes place in less than 400 ms (approximation based on Belgardt et al. [29]). This short evaporation time prevents further movement of the particles to the edge of the droplet.

### Close-Packed Order vs Random Order

**Fig. 6:**
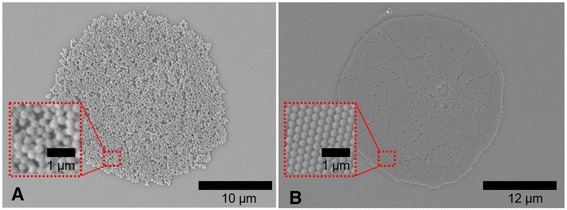
Inkjet-printed deposit with **a** randomly arranged nanospheres obtained by addition of diethylene glycol and **b** close-packed two-dimensional colloidal arrays with hexagonal structure by addition of formamide

In most of the images shown before, the nanospheres are close-packed and highly ordered. Images with higher magnification of the close-packed ordering are available in Additional file 1: Figure S4. Usually, this arrangement of particles is desired and beneficial for many applications in the field of photonics [38], e.g., for optical devices (e.g., Bragg reflectors [35]) and coatings (one example is the application of ordered layers for enhanced light-trapping in solar cells [39]) or structural colors [40]. However, the research about non-close-packed colloidal arrays [41, 42] and disordered photonics, e.g., photonic glasses, has been attracting increasing interest during the last years [43]. Here, not a perfectly ordered arrangement of particles is required but a completely random arrangement allowing a control of the light scattering. Garcia et al. [43] discuss various methods and the incurred difficulties to obtain disordered, random arrangement of nanospheres. So they demonstrate an approach for the manufacturing of disordered layers based on self-assembly by manipulating the colloidal suspensions, e.g., by increased cluster formation of particles through flocculation. In this respect, their approach for the manufacturing of disordered layers of nanospheres as well as ours is based on the manipulation of the colloidal suspension. However, in our case, the suspensions can be deposited by inkjet printing allowing a patterning and to define easily the area and the thickness of the layer. Diethylene glycol (boiling point 245 °C, vapor pressure 0.006 mmHg at 25 °C) was employed as evaporation agent to control the ordering process of the nanospheres. The aqueous ink formulation consisting of 1.5 wt.% of polystyrene particles of BS305 and 6.25 wt.% diethylene glycol was inkjet-printed on HMDS-treated substrates as single droplets. Deposits with randomly arranged nanospheres similar to the photonic glasses reported by Garcia et al. [43] were obtained as shown in Fig. 6a. In contrast, deposits with arrays of remarkably high order in a hexagonally close-packed structure were obtained by adding 2.5 wt.% formamide to the aqueous ink formulation with 1.5 wt.% polystyrene particles of BS305 as shown in Fig. 6b.

## Conclusions

Colloidal suspensions were applied as a model ink in inkjet printing to study the layer formation processes based on self-assembly of nanosphere particles on non-absorbent substrates, which are usually employed in the field of printed electronics. It can be concluded that the three parameters under consideration (1) substrate wettability (the surface energy of the substrate represented by the contact angle with water), (2) the ink formulation, and (3) the temperature of the substrate are very important parameters in inkjet printing defining the morphology of the deposits. By tuning these parameters carefully, desired deposit morphologies for certain applications can be obtained, e.g., deposits with a coffee ring shape, highly ordered monolayer deposits with close-packed colloidal arrays, randomly arranged particles in a droplet deposit and multilayer stacks. Our findings highlight the importance of a tuned interaction between ink, constituents, and substrate surface in inkjet printing and any liquid deposition method towards the formation of micro- and nanoscopically engineered morphologies.

## Additional file

Additional file 1:
**Supporting Information is available online.**
