# Supplementary material for: Inkjet Printing of Colloidal Nanospheres: Engineering the Evaporation-Driven Self-Assembly Process to Form Defined Layer Morphologies
Source: Nanoscale Res Lett. 2015 Sep 16;10:362. doi: 10.1186/s11671-015-1065-2 (PMC4573088; doi:10.1186/s11671-015-1065-2)
Supplement: Additional file 1: — Supporting Information is available online. [file 11671_2015_1065_MOESM1_ESM.docx]

**Supporting Information for**

Inkjet printing of colloidal nanospheres: Engineering the evaporation-driven self-assembly process to form defined layer morphologies

Enrico Sowade,^a*^ Thomas Blaudeck,^a,b*^ Reinhard R. Baumann ^a,c*^

a Technische Universität Chemnitz, Digital Printing and Imaging Technology, 09126 Chemnitz, Germany.

b Technische Universität Chemnitz, Center for Microtechnologies, 09107 Chemnitz, Germany.

c Fraunhofer Institute for Electronic Nanosystems, Department Printed Functionalities, 09126 Chemnitz, Germany.

* corresponding authors: [enrico.sowade@mb.tu-chemnitz.de](mailto:enrico.sowade@mb.tu-chemnitz.de), [thomas.blaudeck@zfm.tu-chemnitz.de](mailto:Thomas.blaudeck@zfm.tu-chemnitz.de), [reinhard.baumann@mb.tu-chemnitz.de](mailto:reinhard.baumann@mb.tu-chemnitz.de)


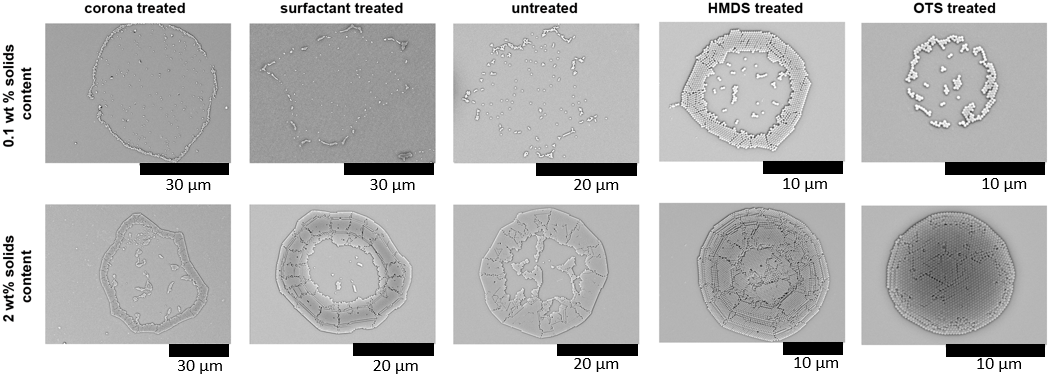


Figure S1: SEM images of the deposits of inkjet-printed droplets of colloidal suspensions with 0.1 wt% solids content and 2 wt% solids content on differently treated glasses; morphology and diameter of the deposits vary according to the treatment


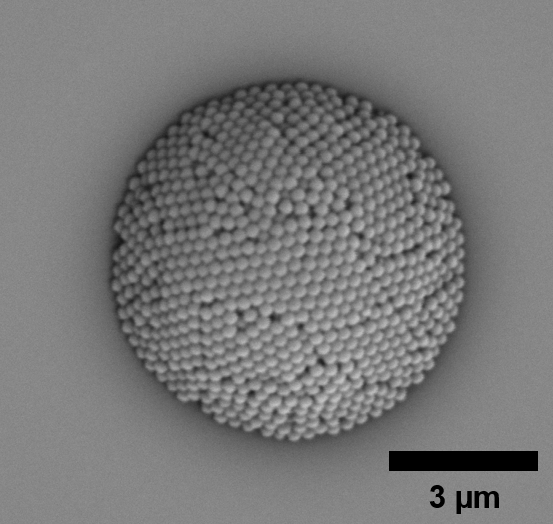


Fig. S2: Inkjet-printed colloidal hemisphere using BS305; the surface shows some line- and point defects of the self-assembled microspheres due to the surface curvature of the hemisphere


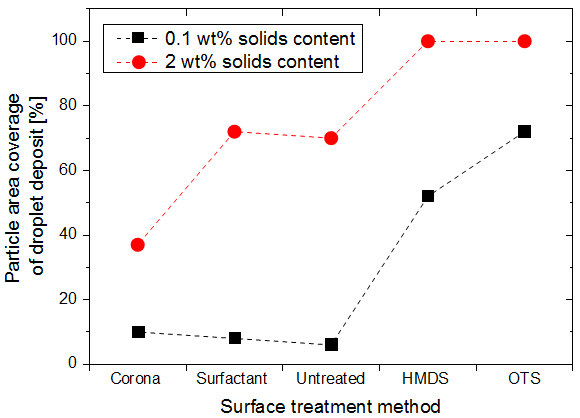


Figure S3: Particle area coverage of representative single droplets as a function of surface treatment method; particle area coverage was calculated by determining the ratio of the number of pixels, which are circumscribed by the particles and the total area (meaning area of particles including the circumscribed area)


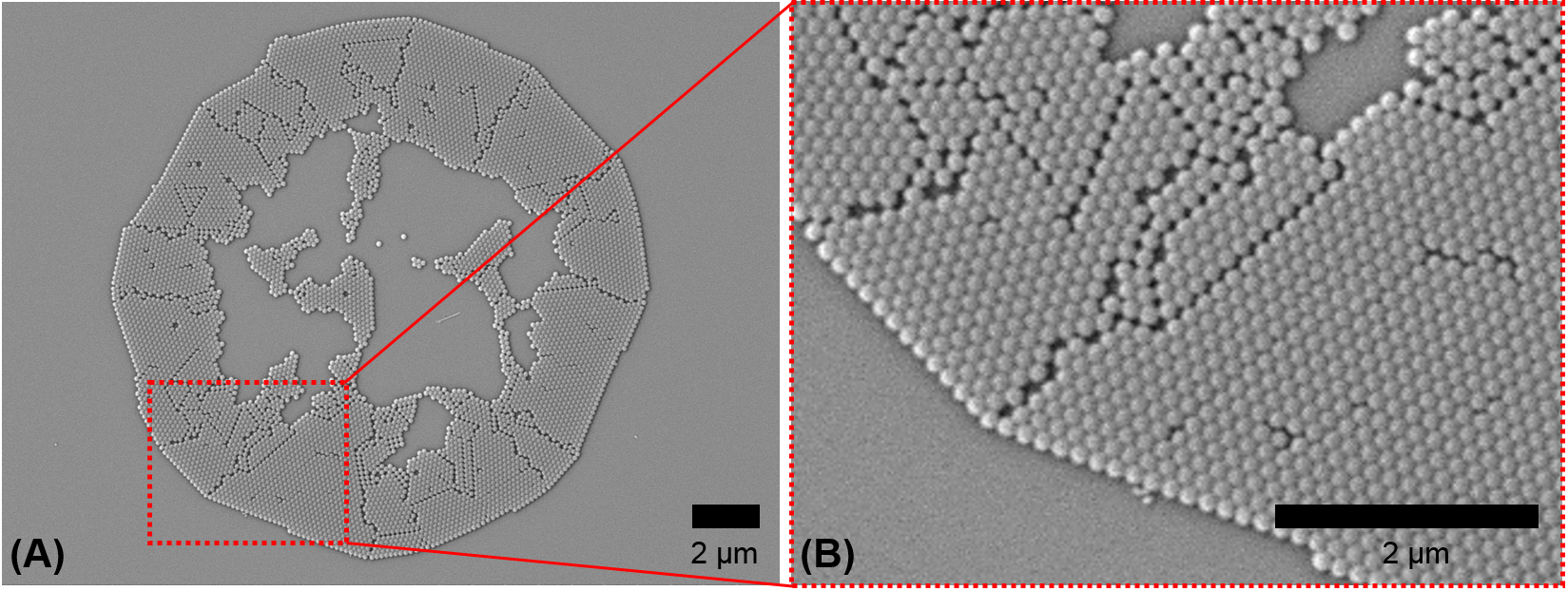


Figure S4: Inkjet-printed deposits with BS305 on cleaned glass, (A) shows the morphology of the single-drop deposit and (B) is a detailed section showing the high ordering of the particles in hexagonal arrays with a few point and line defects
